# Patient-Representing Population's Perceptions of GPT-Generated Versus Standard Emergency Department Discharge Instructions: Randomized Blind Survey Assessment

**DOI:** 10.2196/60336

**Published:** 2024-08-02

**Authors:** Thomas Huang, Conrad Safranek, Vimig Socrates, David Chartash, Donald Wright, Monisha Dilip, Rohit B Sangal, Richard Andrew Taylor

**Affiliations:** 1 Department of Emergency Medicine Yale School of Medicine New Haven, CT United States; 2 Department for Biomedical Informatics and Data Science Yale School of Medicine New Haven, CT United States; 3 Program of Computational Biology and Bioinformatics Yale University New Haven, CT United States; 4 School of Medicine University College Dublin National University of Ireland Dublin Ireland

**Keywords:** machine learning, artificial intelligence, large language models, natural language processing, ChatGPT, discharge instructions, emergency medicine, emergency department, discharge instructions, surveys and questionaries

## Abstract

**Background:**

Discharge instructions are a key form of documentation and patient communication in the time of transition from the emergency department (ED) to home. Discharge instructions are time-consuming and often underprioritized, especially in the ED, leading to discharge delays and possibly impersonal patient instructions. Generative artificial intelligence and large language models (LLMs) offer promising methods of creating high-quality and personalized discharge instructions; however, there exists a gap in understanding patient perspectives of LLM-generated discharge instructions.

**Objective:**

We aimed to assess the use of LLMs such as ChatGPT in synthesizing accurate and patient-accessible discharge instructions in the ED.

**Methods:**

We synthesized 5 unique, fictional ED encounters to emulate real ED encounters that included a diverse set of clinician history, physical notes, and nursing notes. These were passed to GPT-4 in Azure OpenAI Service (Microsoft) to generate LLM-generated discharge instructions. Standard discharge instructions were also generated for each of the 5 unique ED encounters. All GPT-generated and standard discharge instructions were then formatted into standardized after-visit summary documents. These after-visit summaries containing either GPT-generated or standard discharge instructions were randomly and blindly administered to Amazon MTurk respondents representing patient populations through Amazon MTurk Survey Distribution. Discharge instructions were assessed based on metrics of *interpretability of significance*, *understandability*, and *satisfaction*.

**Results:**

Our findings revealed that survey respondents’ perspectives regarding GPT-generated and standard discharge instructions were significantly (*P*=.01) more favorable toward GPT-generated return precautions, and all other sections were considered noninferior to standard discharge instructions. Of the 156 survey respondents, GPT-generated discharge instructions were assigned favorable ratings, “agree” and “strongly agree,” more frequently along the metric of *interpretability of significance* in discharge instruction subsections regarding diagnosis, procedures, treatment, post-ED medications or any changes to medications, and return precautions. Survey respondents found GPT-generated instructions to be more *understandable* when rating procedures, treatment, post-ED medications or medication changes, post-ED follow-up, and return precautions. *Satisfaction* with GPT-generated discharge instruction subsections was the most favorable in procedures, treatment, post-ED medications or medication changes, and return precautions. Wilcoxon rank-sum test of Likert responses revealed significant differences (*P*=.01) in the *interpretability of significant* return precautions in GPT-generated discharge instructions compared to standard discharge instructions but not for other evaluation metrics and discharge instruction subsections.

**Conclusions:**

This study demonstrates the potential for LLMs such as ChatGPT to act as a method of augmenting current documentation workflows in the ED to reduce the documentation burden of physicians. The ability of LLMs to provide tailored instructions for patients by improving readability and making instructions more applicable to patients could improve upon the methods of communication that currently exist.

## Introduction

Discharge instructions serve as an essential bridge between hospital treatment and at-home recovery. Particularly in the emergency department (ED), where clinical team members have limited time to review the events of the ED encounter and follow-up recommendations with their patients, discharge instructions are critical to communicating information to patients and increasing adherence to follow-up care. Specifically, discharge instructions serve to inform the patient about key details such as their diagnosis, evaluations performed, preliminary diagnostic test results, medications, treatment plans, follow-up care, and reasons to return to the ED [1,2]. Improved patient understanding of discharge instructions and self-efficacy following discharge have been linked to improved health outcomes and decreased readmission rates [3-5]. Despite their important role, many patients leave the hospital with discharge instructions that are jargon filled and difficult to navigate, with studies showing that up to 88% of discharge instructions are unreadable to the population served [6-8]. General guidelines suggest that all health-related information provided by a physician to patients should be readable at a sixth-grade reading level [2,9]. Furthermore, the lack of personalization in many current discharge instructions misses an opportunity to better engage patients with understanding their care and diagnosis, which has been shown to be key to increasing patient health outcomes [10,11]. This communication gap drives nonadherence and higher rates of patient readmission [12,13]. Addressing this challenge of bridging physician and patient discharge communication is essential for improving patient health outcomes and the overall effectiveness of care delivery.

The recent advancement of large language models (LLMs), such as ChatGPT, offers a potential solution to the longstanding issue of inaccessible medical communication and the time-demanding nature of providing care in the ED [14,15]. Research on LLMs has demonstrated their broad capabilities in medical question answering, including passing the United States Medical Licensing Examinations [16]. These models can generate medical text that is accurate, informative, more readable, and even more empathetic [17-21]. By leveraging LLMs, there is the potential to create discharge instructions that are not only tailored to individual patients but also presented in a format that is engaging and easy to understand.

Recent research has underscored the feasibility of using LLMs for patient discharge instructions. The translation of 50 patient discharge summaries from the medical sublanguage to regular English by GPT-4 demonstrated significant improvement in objective readability scores [21]. This study also showed these LLM-generated summaries had a generally acceptable level of accuracy and completeness, per physician assessment. Other research has extended to specialties such as neurology and radiology, demonstrating that LLMs can digest complex medical text and produce summaries that are patient-centric and sufficiently comprehensive that physicians are comfortable releasing them to patients [22-24].

While this existing LLM research has laid a robust foundation, it has so far lacked a key evaluative component: integration of patient perspectives in a randomized unbiased comparison of LLM-generated text against the current standard of care. In addition, current investigations of leveraging LLMs to generate discharge instructions or summaries in the ED remain limited. We aim to address this gap by surveying a patient-representing population using a randomized blinded approach to compare LLM-generated, fully formatted discharge instructions to the standard discharge instructions.

## Methods

### Study Population and Setting

The study population was a convenience sample of adult patients (aged ≥18 years) taken from Amazon MTurk from December 31, 2023, to March 31, 2024; residing in the United States; with an approval rate (an indicator in Amazon MTurk of work quality) of >90%; and who were also categorized as Amazon MTurk Masters [25]. Research has shown that the majority of patients do not have medical training, have low health literacy, and have difficulty understanding medical jargon [26,27]. Thus, exclusion criteria for this population included respondents having any significant health care training background to best represent the general patient population. No Amazon MTurk survey respondents were allowed to repeat the survey; therefore, all responses are from unique respondents, reducing bias from individual responders and improving data quality.

### Ethical Considerations

This study was approved by the institutional review board as an exemption (HIC #2000036301). Informed consent was obtained at the initiation of the survey. If consent was declined, the survey ended immediately. Participants were allowed to opt out of the survey at any time, and survey data only used fully completed surveys. Data were anonymized and the Amazon MTurk ID was used only for payment but not used for analysis. Participants were compensated US $2.62 for their participation if they completed the survey and met the basic quality control and attention check metrics. These details were clearly outlined in both the Amazon MTurk survey distribution description as well as the initial informed consent.

### Synthesizing Fictional ED Encounters

A set of synthetic fictional ED notes were generated that represent 5 separate independent ED encounters (Multimedia Appendix 1). These notes were diverse in patients’ age, sex, ethnicity, and clinical presentation. All ED notes were authored by an emergency medicine (EM) attending physician (DW) to emulate realistic ED settings and were rendered in multiple document styles. The EM physician note included a brief chief complaint statement, history of present illness, past medical history, physical examination, and assessment and plan. The nursing note contained summaries of the presentation and care performed.

### ChatGPT Prompt Development

Initial prompts were developed based on the Society for Academic Emergency Medicine and Joint Commission guidelines for discharge instructions [1,2]. Prompts were applied to the 5 synthetic clinical note sets used for the final surveys (the final GPT prompt is available in Multimedia Appendix 1). Slight adjustments were made iteratively to improve the GPT-generated discharge instruction output, including changes the organization provided in the prompt, additional directive phrasing, and evaluation of output. Final prompts added to the synthetic clinical notes described above were deployed via a pipeline to automatically query and retrieve responses from OpenAI’s GPT-4 for the final 5 clinical scenarios. Other strategies described in previous prompt engineering work were also included in this iterative process to improve GPT-4 recognition of the note and generative responses [28].

### Discharge Instructions Development

Each clinical scenario represented by the synthetic clinical note was passed to GPT-4 with the final prompt generated by the methodology described above. Each of the 5 clinical scenarios was associated with 2 sets of discharge instructions for a total of 10 different variants of discharge instructions. These discharge instructions consist of 1 GPT-4–generated discharge instruction and 1 standard discharge instruction a patient would expect to receive from the ED without GPT-4–generated text. The standard discharge instructions (provided by RBS) are the standardized Epic After Visit Summary with complaint-specific discharge instructions from 1 of 2 institutionally contracted patient education resources (Elsevier Patient Education and UpToDate). These discharge instructions were then populated, through Epic’s “Playground” environment, into a generated ED encounter so that an entire discharge instruction could be generated with the same overall formatting. The standard discharge instructions did not include physician-generated free text. All specific identifiers, such as phone numbers, insurance names, and trademarks, were censored out of the final generated discharge instructions.

### Development, Recruitment, and Distribution

Surveys were created in Qualtrics to be distributed through the distribution service within Amazon MTurk, a reliable and widely used survey distribution service (the Qualtrics survey is available in Multimedia Appendix 1). These surveys were iteratively reviewed by ED physicians, residents, and medical students to ensure readability and ease of use. The survey was an open survey format, available to all Amazon MTurk Masters who also had an approval rating of >90%. The survey was listed on Amazon MTurk and was only visible to MTurkers that met the requirements. No other forms of advertising occurred other than the listing on Amazon MTurk.

### Survey Administration

**Figure 1:**
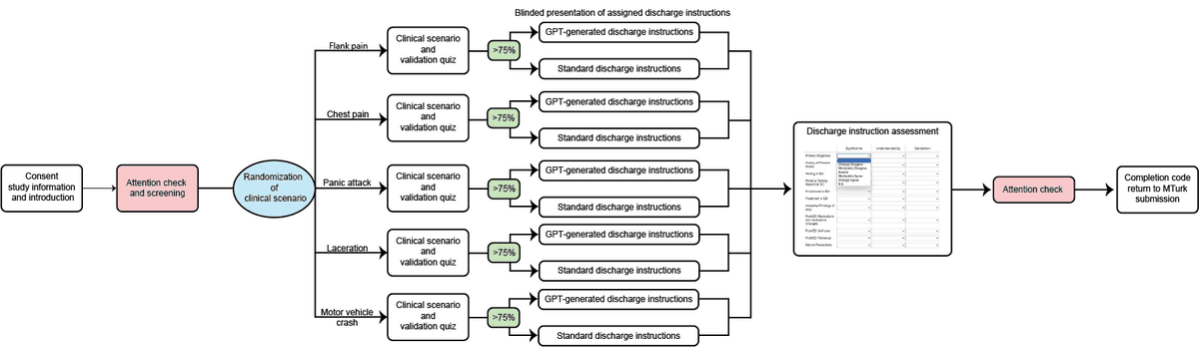
The Qualtrics survey design for discharge instruction randomized blind assessment by Amazon MTurk respondents. Survey respondents first passed an initial consent documentation, a screener for health care or medical background, and a series of attention checks. Respondents were then randomized to 1 of 5 possible clinical scenario and then randomized to view either the GPT-generated or standard version of discharge instructions. This respondent was then tasked to answer Likert-scale questions regarding the 3 metrics: interpretability of significance, understandability, and satisfaction, for each discharge instructions subsection, before 1 final attention check and the conclusion of the survey.

The survey was distributed on Amazon MTurk over a 3-month period, during which respondents were given a link to the complete survey built on Qualtrics. The survey structure included an initial explanation of what the survey responder would be presented with—1 clinical scenario described by a note that contains the pertinent information to the ED visit. The survey flow saw each respondent blindly randomized to 1 of 10 possible workflows. The respondent was initially presented with consent documentation, initial screening questions, and attention checks that must be passed for survey results to be accepted [29,30]. Respondents were then randomized to 1 of 5 random clinical scenarios where they responded to an additional quality control quiz including 4 multiple choice questions regarding the clinical scenario (Figure 1).

The survey respondent was then tasked to fill out a matrix of Likert-scale questions regarding the discharge instructions they read in the context of the clinical scenario (the clinical scenarios and discharge instructions are available in Multimedia Appendix 1). The y-axis included all components of the discharge instruction: diagnosis, history of the current problem or illness, testing received in the ED, any pending test results, procedures, treatment, incidental findings, post-ED medications or any changes to medications, post-ED self-care, post-ED follow-up, and return precautions. The x-axis of the matrix asked the survey respondent to rate on a traditional Likert scale on the 3 metrics of interpretability of significant findings or information, ease of understanding, and satisfaction concerning each of the discharge instruction subsections (Multimedia Appendix 2).

Attention checks and quality checks were included throughout the survey to ensure attention and increase the likelihood of high-quality data. Attention checks, such as asking the patient to answer simple but targeted questions with expected correct answers, are a method of ensuring survey respondents are reading questions and responding to the best of their ability [31]. A manual review of each round of distribution was performed to maintain data quality by assessing the percentage of attention checks passed per survey respondent and validating respondent understanding of the clinical scenario through a passing score (≥3/4, ≥75%) on the corresponding quiz. Respondents were allowed to go back to change their answers when necessary. Survey respondents’ results were only retained for the final data set if they passed the content validation test (≥3/4, ≥75%) and failed no more than 1 of 4 attention checks to ensure a high-quality data set.

### Data Analysis

Survey responses were extracted from Qualtrics and analyzed using Python (version 3.11.5; Python Software Foundation). Frequency counts of Likert responses to each metric and discharge instruction subsection were organized and visualized in clustered stacked multi-bar charts. The Wilcoxon rank-sum test was used to analyze for significant differences between Likert responses between GPT-generated and standard discharge instructions.

## Results

### Overview

**Figure 2:**
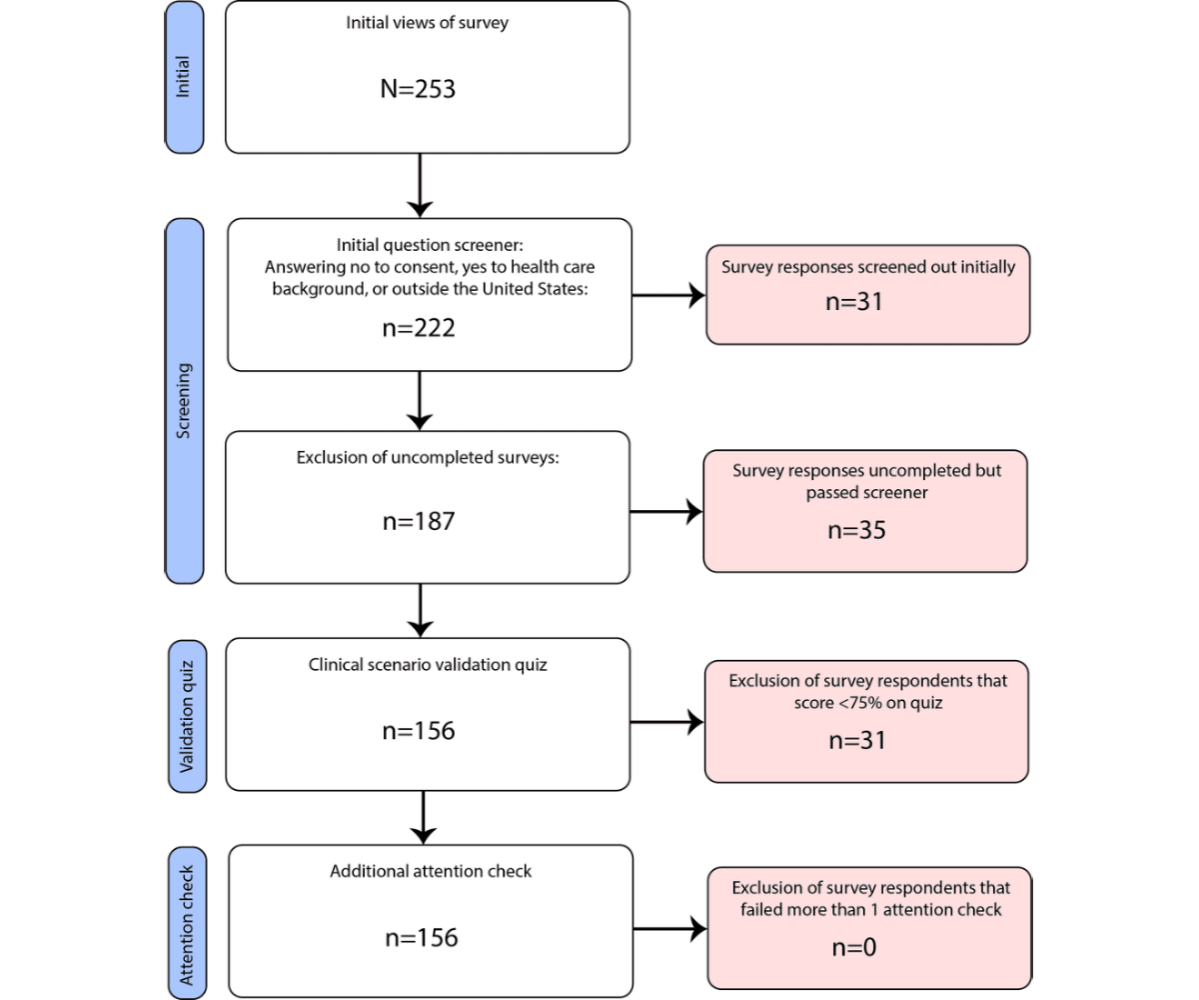
The stepwise method toward exclusion of survey respondents. From an initial 253 views of the survey, 222 were not screened out by the initial screen of consent, not having a health care background, and not residing in the United States. Following the initial screener, 35 did not finish the entire survey. Surveys also had their validation quiz about the clinical scenario and attention checks graded. Of 187 completed surveys, 156 passed the validation quiz, and all respondents who passed the validation quiz also successfully answered all other attention checks correctly.

From 253 unique initial views on Amazon MTurk, 222 (87.7%) passed the initial screening criteria. Of those 222 respondents, 187 (84.2%) respondents successfully completed the survey, and 156 (83.4%) of those 187 respondents passed all validation and attention checks, making them eligible for the final cohort (Figure 2).

### Domain 1: Interpretability of Significance

To assess the interpretability of the significance of the discharge instructions, each survey respondent was tasked to rate their agreement with the statement: “the information in the discharge instruction (subsection) effectively explains the interpretability of significance of the findings in a way that’s personalized to me (the hypothetical recipient) and is easy to follow.” They were asked to individually assess the interpretability of significance regarding each important subsection of the discharge instructions. Of note, the frequency of “agree” and “strongly agree” selected by survey respondents was greater across all GPT-generated discharge instructions’ subsections with regard to the interpretability of significance except for pending test results and incidental findings.

**Figure 3:**
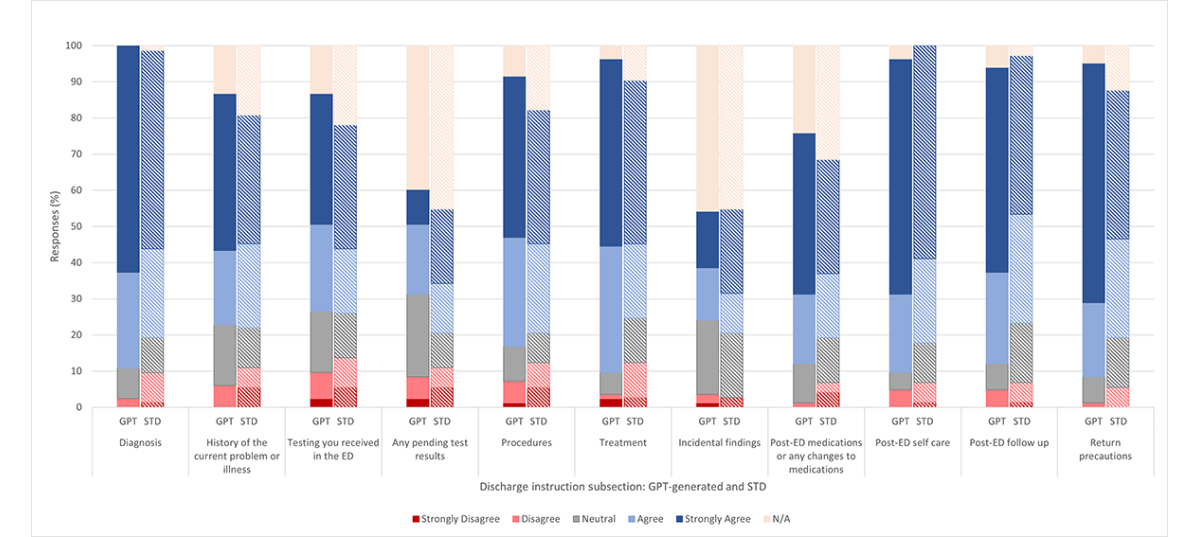
Agreement regarding each subsection of the discharge instructions about the prompt: “the information in the discharge instruction (subsection) effectively explains the significance of the findings in a way that’s personalized to me (the hypothetical recipient) and is easy to follow.” ED: emergency department; STD: standard.

Regarding “pending test results,” 29% (24/83) of respondents rated favorably for their ability to interpret significant information in GPT-generated discharge instructions and 34% (25/73) rated favorably in standard discharge instructions. For incidental findings, 30% (25/83) and 34% (25/73) of respondents rated favorably for GPT-generated and standard discharge instructions, respectively. These 2 sections saw the greatest percentage of not applicable responses as well, with 39% (32/83) versus 45% (33/73) in pending testing results and 45% (37/83) versus 45% (33/73) for incidental findings, respectively, for GPT-generated and standard discharge instructions (Figure 3).

All other subsections of the GPT-generated discharge instructions were scored more favorably in terms of the interpretability of significance metric, with the most notable difference coming from interpreting the significance of diagnosis (GPT: 74/83, 89% vs standard: 58/73, 80%), procedures (GPT: 62/83, 75% vs standard: 45/73, 62%), treatment (GPT: 72/83, 87% vs standard: 48/73, 66%), post-ED medications or any changes to medications (GPT: 53/83, 64% vs standard: 36/73, 49%), and return precautions (GPT: 72/83, 87% vs standard: 50/73, 68%). However, these differences in ratings between GPT-generated and standard discharge instructions were not statistically significant (Table 1). Differences in average Likert score as graded on a numerical scale for the interpretability of significance are available in Multimedia Appendix 3.

**Table 1:** The results of the Wilcoxon rank-sum test between GPT and standard discharge instructions across all 3 metrics of interpretability of significance, understandability, and satisfaction on a traditional Likert scale.

Discharge instruction subsection	Significance		Understandability		Satisfaction	
	Statistic	*P* value	Statistic	*P* value	Statistic	*P* value
Diagnosis	1.0889	.28	1.0288	.30	0.0799	.94
History of the current problem or illness	0.6777	.50	–0.5204	.60	0.4410	.66
Testing you received in the ED^a^	0.1992	.84	–0.4277	.67	–1.0055	.31
Any pending test results	–1.3601	.17	–1.1275	.26	–1.0330	.30
Procedures	0.7495	.45	1.1351	.26	1.1768	.24
Treatment	1.1914	.23	0.9122	.36	0.6776	.50
Incidental findings	–0.9949	.32	–0.9091	.36	–0.4280	.67
Post-ED medications or changes	1.5435	.12	0.4388	.66	1.0104	.31
Post-ED self-care	1.0812	.28	1.1214	.26	0.1954	.85
Post-ED follow-up	1.8320	.07	1.6280	.1035	0.8989	.37
Return precautions	2.5617	.01	1.4151	.16	1.9154	.06

^a^ED: emergency department.

### Domain 2: Understanding

The understanding was assessed by their agreement with the phrase: “the information in the discharge instruction (subsection) is written such that it is presented in a clear and straightforward manner that is easily comprehensible.” Although not statistically significantly different, there are some differences to note between the discharge instructions authored by GPT compared to the standard discharge instructions from the ED (Table 1).

**Figure 4:**
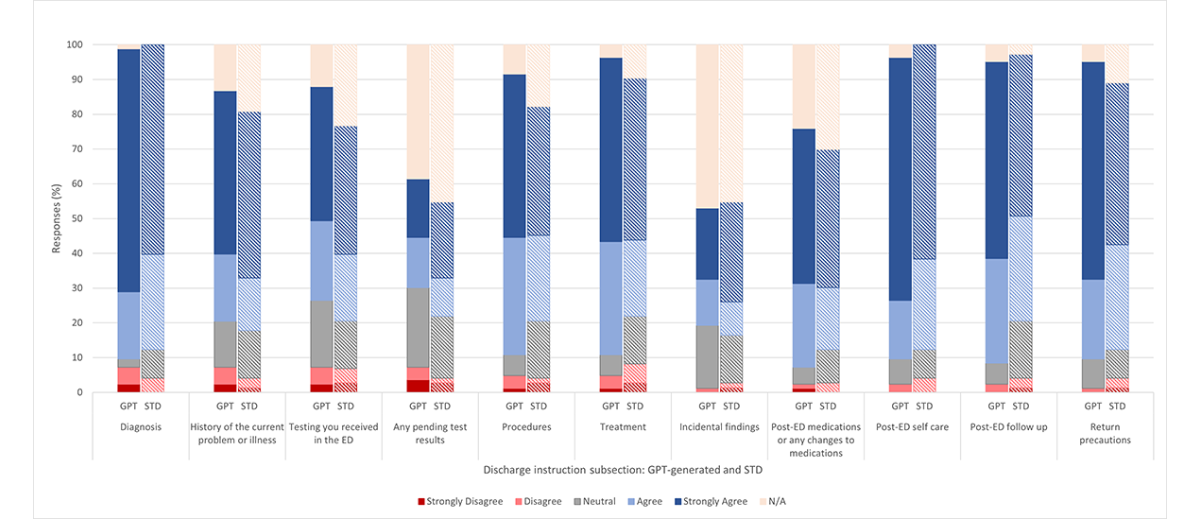
This is a clustered stacked multibar chart of the 5 possible ratings (strongly disagree, disagree, neutral, agree, and strongly agree) regarding each subsection of the discharge instructions to assess understanding concerning the prompt: "the information in the discharge instruction (subsection) is written such that it is presented clearly and straightforwardly that is easily comprehensible." ED: emergency department; STD: standard.

While survey respondents recorded a greater interpretation of significance from diagnosis, their understanding was very similar between GPT (74/83, 89.2% agree or greatly agree) and standard discharge instructions (64/73, 87.6%). However, the other subcategories that saw a greater percentage of survey respondents rating their understanding of the information in specific discharge instruction subsections favorably came from procedures (GPT: 67/83, 81% vs standard: 45/73, 62%), treatment (GPT: 71/83, 86% vs standard: 50/73, 68%), post-ED medications or medication changes (GPT: 57/83, 69% vs standard: 42/73, 58%), post-ED follow-up (GPT: 72/83, 87% vs standard: 56/73, 77%), and return precautions (GPT: 71/83, 86% vs standard: 56/73, 77%; Figure 4). It is important to note, however, that these differences did not achieve statistically significant differences (Table 1). Differences in average Likert score as graded on a numerical scale for understandability are available in Multimedia Appendix 4.

### Domain 3: Satisfaction

**Figure 5:**
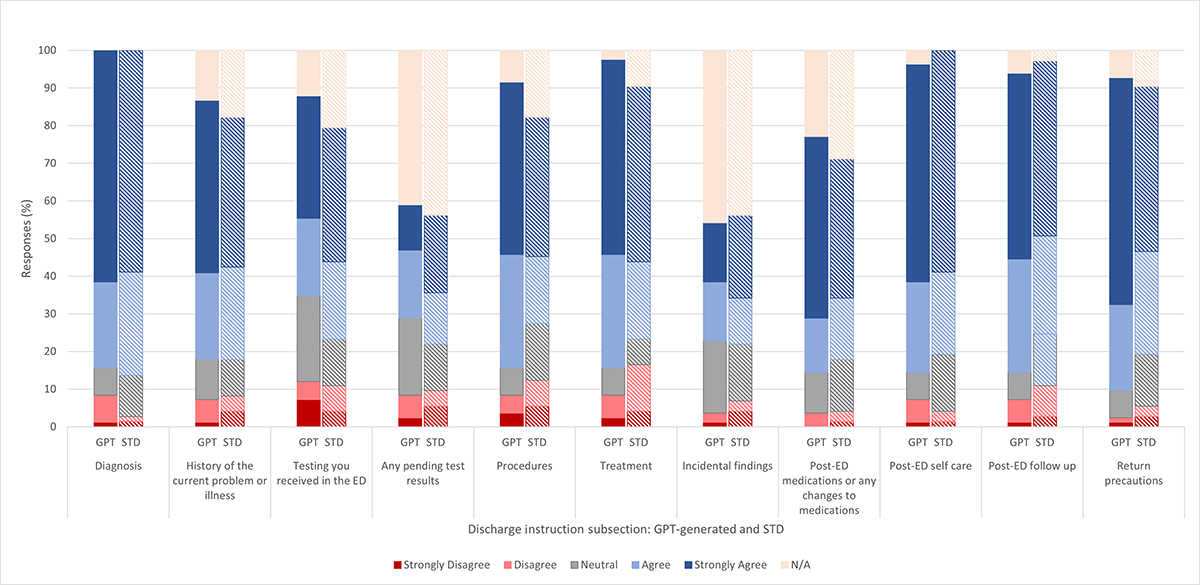
This is a clustered stacked multibar chart of the five possible ratings (strongly disagree, disagree, neutral, agree, and strongly agree) regarding each subsection of the discharge instructions to assess satisfaction in relation to the prompt: "the information in the discharge instructions (subsection) fulfill your personal expectations of the quality you would expect to receive in an ED setting." ED: emergency department; STD: standard.

Satisfaction was assessed based on their agreement with the phrase: :the information in the discharge instructions (subsection) fulfills your personal expectations of the quality you would expect to receive in an ED setting.: Within satisfaction, the GPT-generated discharge instructions once again saw greater frequency of agree and strongly agree across the majority of discharge instruction subsections, signaling positive sentiment toward GPT-generated discharge instructions in comparison to the standard discharge instructions (Figure 5). A greater percentage of survey respondents rated their satisfaction with GPT-generated discharge instruction subsections the most favorably in procedures (GPT: 63/83, 76% vs standard: 40/73, 55%), treatment (GPT: 71/83, 86% vs standard: 50/73, 68%), post-ED medications or medication changes (GPT: 52/83, 63% vs standard: 39/73, 53%), and return precautions (GPT: 69/83, 83% vs standard: 52/73, 71%).

However, there was no statistically significant difference between the ratings regarding GPT-generated and standard discharge instructions (Table 1). The sections in which survey respondents were slightly more dissatisfied with GPT-generated discharge instructions were tests received in the ED (GPT: 44/83, 53% vs standard: 41/73, 56%; rated satisfied or very satisfied), pending test results (GPT: 25/83, 30% vs standard: 25/73, 34%), and incidental findings (GPT: 26/83, 31% vs standard: 25/73, 34%). Differences in average Likert score as graded on a numerical scale for satisfaction are available in Multimedia Appendix 5.

### Comparative Analysis

We conducted a Wilcoxon rank-sum test to compare the Likert results across GPT and standard discharge instruction cohorts. Due to the nonparametric, ordinal nature of the data, the Wilcoxon rank-sum test was best suited to better understand the results.

Notably, the interpretability of significant findings or information was considered significantly (*P*=.01) more favorable in GPT-generated return precautions compared to standard discharge instructions. No other specific subsection of the discharge instructions was rated by respondents to statistically significantly differ between the GPT-generated and standard discharge instructions on a standard Likert scale (Table 1). Other discharge instruction subsections were generally positive, indicating a slight but not significant favorability toward GPT-generated discharge instructions (Table 1). The exceptions were consistent negative test statistics in pending test results and incidental findings across all 3 metrics.

## Discussion

### Principal Findings

We present a novel assessment of a general patient population’s perception of discharge instructions, a key method of communication between patients and physicians. Although the GPT-generated discharge instructions received higher ratings across all 3 metrics of interpretability of significant information, understandability, and satisfaction in several key subsections of the discharge instructions, the standard discharge instructions were not found to be statistically significantly different from the discharge instructions authored by a LLM (GPT-4) except for the interpretability of significant information in return precautions (Table 1). The GPT-generated return precautions frequently included personalized and specific information, such as symptoms to be aware of or progression of current symptoms from the current presentation, corresponding to the primary diagnosis from the ED encounter scenario. Prior literature demonstrates that return precautions are oftentimes one of the most crucial discharge instruction subsections and the section that has the greatest deficit in recall and understanding [32]. This personalized and clearly presented information demonstrates 1 method in which GPT-generated discharge instructions could serve as an adjunct for the current workflow within discharge summaries to help improve patient understanding and interpretation of key information.

The similar and even slightly higher ratings for GPT-generated discharge instructions suggest the potential for LLMs to be able to serve as possible adjuncts or interventions in improving discharge instructions or throughput in the ED. LLMs have the ability to generate not only accurate but also detailed and personalized discharge instructions that may improve patients’ abilities to interpret the significance of complex medical information through simplification, which can not only play an important role in reducing documentation burden for the physicians but also possibly ease patient transitions out of the hospital through augmented and possibly improved forms of documentation and medical information [33,34].

Despite the lack of statistically significant superiority for most metrics and subsections, the performance of LLM-generated discharge instructions compared to standard methods suggests that the former are not inferior either. Several subsections of the GPT-generated discharge instructions were even rated favorably in comparison to the standard discharge instructions. Notably, information regarding procedures and treatments received in the ED, as well as important follow-up information including post-ED medication or medication changes and return precautions, had a greater frequency of favorable ratings across all 3 metrics, interpretability of significance, understandability, and satisfaction, as compared to standard discharge instructions. This equivalence opens up practical applications for LLMs in the discharge process, particularly in terms of efficiency and reducing clinician workload. EDs often have bottlenecks in patient flow that may benefit from LLM integration to expedite the discharge process. Since LLM-generated instructions can be produced rapidly and tailored to individual patient profiles, they have the potential to decrease the time clinicians spend writing these instructions, and there may be value in reducing the discharge-to-departure time [34,35]. Providing a method to quickly author initial drafts or starting points for discharge instructions may free up clinician time for other high-value tasks. LLMs had trending higher scores post-ED care and follow-up, which often have to be customized for each patient. By providing an initial starting point for writing discharge instructions that already include detailed pertinent information that can pull in prior notes and potentially other data fields in future implementations, ChatGPT generation of notes has the potential to reduce documentation burden and lead to improved and more personalized discharge instructions. Furthermore, the adaptability of LLMs could lead to more dynamic discharge instructions. For instance, discharge instructions could be automatically updated as soon as a clinician finalizes their notes, ensuring that patients receive the most current and relevant information without delay.

### Comparison to Prior Work

Prior methods of generating fictional cases, specifically in orthopedics, and synthesizing discharge documentation by ChatGPT in comparison to a standard showed that they were comparable in quality as assessed by expert panels [36]. Our study found similar results, with patient-representing survey respondents finding that discharge instructions generated using ED synthetic notes were of similar quality to standard ED discharge instructions. Barak-Corren et al [14] evaluated pediatric EM attending perceptions regarding summaries regarding completeness, accuracy, efficiency, readability, and overall satisfaction. Our findings represented similar results from the patient perspective, with survey respondents assessing GPT-generated discharge instructions with greater positive sentiment than negative sentiment.

### Limitations

This study poses several limitations. The participants recruited in this study were compensated to answer to their best ability in these surveys. They were asked to assume the role of a patient in an ED, receive information regarding a clinical scenario, and respond as if they were a patient receiving the corresponding discharge instructions. Survey respondents were asked to complete rigorous attention checks and quality control metrics to ensure they properly understood the clinical scenario, but it is possible that survey respondents did not accurately assess the discharge instructions following the attention checks and other quality control metrics. The task presented to survey respondents was not simple and was a complex task.

The generated discharge instructions were improved through an iterative prompt engineering process that may have assessed and improved quality specifically for the ED setting. Due to the use of synthetic notes in this pipeline, the generalizability of these methods may be limited based on the specialty of discharge instructions as well as important variations of discharge instructions from provider to provider. The discharge instructions were also generated from documentation such as the provider history and physical, which may not consistently be documentation that is fully finished by the time discharge instructions are written. In addition, the prompt development occurred over the same set of notes and clinical scenarios that were used for the final discharge instructions. These limitations may act as barriers to the implementation of the proposed pipeline in current workflows in the ED.

In addition, it is important to note that Amazon MTurk survey respondents are not the same subpopulation presenting to EDs, only a convenience sample representative of the general population. However, the demographics of Amazon MTurk survey respondents are similar to that of patients presenting to EDs in select features in Connecticut, which is considered to be one of the most representative states of the United States [37]. Notably, Amazon MTurk survey respondents are 57% female vs 58% female among ED presentations. Annual household incomes among Amazon MTurk survey respondents also closely resemble the general US population in income ranges under US $150,000 per the Current Population Survey. For income ranges greater than US $150,000, only 4.92% of MTurkers fall within this range while Current Population Survey reports 15.47% for the generation population. However, Amazon MTurk survey respondents skew younger compared to ED presentations, with 66.5% of Amazon MTurk survey respondents aged between 18 and 40 years and less than 40% of ED presentations aged between 18 and 44 years [38]. Survey respondents slightly overrepresent White non-Hispanic populations with approximately 79.9% compared to 66% of ED presentations of being White non-Hispanic in Connecticut. In addition, Amazon MTurk survey respondents are historically better educated compared to the general population, an important consideration when assessing the readability of discharge instructions [39]. Although not perfectly representative, the responses and perspectives of Amazon MTurkers are still important to consider as potential stakeholders in the reception of discharge instructions and a unique perspective external to physician and medical team perspectives. The MTurk population has been shown to be more representative of the general population than any other web-based survey and consistently produces reliable results [40]. However, it is still important to consider replicating similar methods with real patient populations in the ED.

### Future Directions

Our study explored the initial stages of patient-representing perceptions of using LLM-generated discharge instructions in the ED setting. Due to initial constraints on protected health information, synthetic clinical scenarios, clinician notes, and nursing notes were used in developing discharge instructions in this survey format. Future work can focus on the integration of LLMs into existing EHR infrastructure to leverage real patient notes with Health Insurance Portability and Accountability Act–compliant methods of ChatGPT such as through Doximity GPT or the Azure OpenAI system.

The current scope of our work only looked into 5 different presenting chief concerns due to the synthetic note constraint. The next steps could look toward expanding the scope of the use of LLMs in discharge instructions, such as integrating multicultural and multilingual personalization into discharge instructions as well as expanding the amount of teaching and patient education embedded into the discharge instructions [41,42]. The iterative process of GPT-4-based discharge instruction development can also be improved with embedding methods of patient feedback, such as structured interviews, as demonstrated in prior research of other forms of discharge instructions that could further help develop an improved generation of tailored discharge instructions [43].

### Conclusions

The results of this study indicate the promising capabilities for generative LLMs such as GPT-4 in improving methods of health care communication. Future research in the use of LLMs in ED workflows can use this in real-world applications to assess the perceptions of LLM-generated medical documentation by health care staff as well as a possible survey distribution within EDs and real clinical settings that our survey distribution aimed to imitate.

## Appendix

Multimedia Appendix 1 Clinical scenarios and GPT-generated and standard discharge instructions, ChatGPT prompt used to generate the GPT-generated discharge instructions, and Qualtrics survey.

Multimedia Appendix 2 Respondents to the Likert-style matrix survey were tasked to answer with regard to 3 metrics: interpretability of significance, understandability, and satisfaction for the discharge instruction subsection. ED: emergency department.

Multimedia Appendix 3 Differences in averages of Likert scores between GPT and standard discharge instructions for the evaluation of interpretability of significance by discharge instruction subsection.

Multimedia Appendix 4 Differences in averages of Likert scores between GPT and standard discharge instructions for the evaluation of ease of understanding by discharge instruction subsection.

Multimedia Appendix 5 Differences in averages of Likert scores between GPT and standard discharge instructions for the evaluation of satisfaction by discharge instruction subsection.

## Abbreviations

ED: emergency department
EM: emergency medicine
LLM: large language model
