# Supplementary material for: Patient-Representing Population's Perceptions of GPT-Generated Versus Standard Emergency Department Discharge Instructions: Randomized Blind Survey Assessment
Source: J Med Internet Res. 2024 Aug 2;26:e60336. doi: 10.2196/60336 (PMC11329854; doi:10.2196/60336)
Supplement: Multimedia Appendix 1 [file jmir_v26i1e60336_app1.docx]

**Multimedia Appendix 1.**

**Section 1.** Repository of Clinical Scenario Clinician and Nursing Notes, GPT-generated and Standard Discharge Instructions

[Discharge Instructions Survey Notes](https://yaleedu-my.sharepoint.com/:f:/g/personal/t_huang_yale_edu/Emm0XEAoDLdDltPlhMrUIpABy68JotSM6lICY7DG7q1H1Q?e=YWBitr)

**Section 2.** Final ChatGPT Prompts used to generate the final set of discharge instructions.

I am an ED physician and need help writing discharge instructions for my patient, Mr. John Smith. Our EHR system automatically generates basic discharge instructions that cover the standard pertinent material (visit date, medication list, medication changes, procedures performed, incidental findings, f/u instructions etc.); However, I need help writing the "manual" supplemental field, which is the personalized part of the discharge instructions highlighting the most important instructions and information regarding my patients diagnosis and necessary ongoing treatment/follow-up, particularly information that may not be present in the automatically generated discharge instructions.

Based on the encounter notes below, please write the manual personalized discharge instruction section to supplement the automatically generated discharge note. Please make sure it is personalized to the patient, interesting to read, and covers pertinent information very succinctly. No need for too much preamble please jump straight into the most pertinent information. Please provide some personalized patient education for any key diagnoses or incidental findings. Make the note readable to a patient with high school education. However, remember you are a physician and keep a formal and serious but informative tone. Be sure to include the following subsections of the discharge instruction, IF the information is available from the encounter notes below:

1. Primary Diagnosis
2. History of Present Illness
3. Testing in the ED (if any)
4. Pending Testing Results upon Discharge (if any)
5. Procedures received in the emergency department (if any)
6. Treatment received in emergency department (if any)
7. Incidental Findings (if any)
8. Post-emergency department medications and medication changes (if any)
9. These changes include new medications, changes in dosage, or discontinuation
10. Post-emergency department follow-up Instructions/Advice
11. Further investigation if necessary
12. Planned investigations, whether and where these investigations will occur
13. Contact or make appointment with PCP if applicable
14. Return Precautions
15. What red flags to return to the ED for
16. Specify time frame in which patient should return to the ED

Most importantly, I feel like my patients never finish reading all the important info in the full discharge instructions… To combat this, please make the manual discharge note portion concise and to the point. Use subsections, headers, and bulleted lists for ease of reading. Make sure to highlight key information that my patient might gloss over in the automatically generated discharge instructions. Remember to keep it professional.

**Section 3.** Qualtrics Survey Link to the Discharge Instructions Survey Distributed on Amazon MTurk:

<https://yalesurvey.ca1.qualtrics.com/jfe/form/SV_e9VD90YRhaTKLlk>
