# Supplementary figures and images for: Patient-Representing Population's Perceptions of GPT-Generated Versus Standard Emergency Department Discharge Instructions: Randomized Blind Survey Assessment
Source: J Med Internet Res. 2024 Aug 2;26:e60336. doi: 10.2196/60336 (PMC11329854; doi:10.2196/60336)

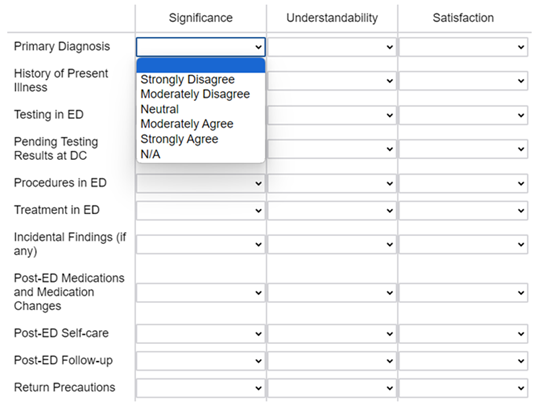

Supplement: Multimedia Appendix 2 [file jmir_v26i1e60336_app2.png]

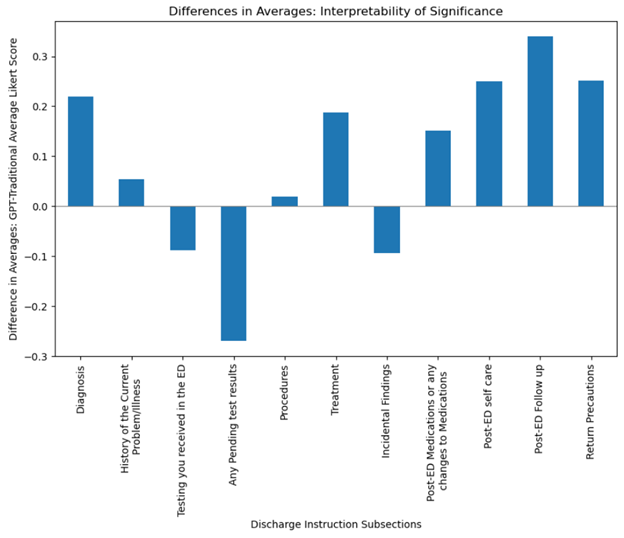

Supplement: Multimedia Appendix 3 [file jmir_v26i1e60336_app3.png]

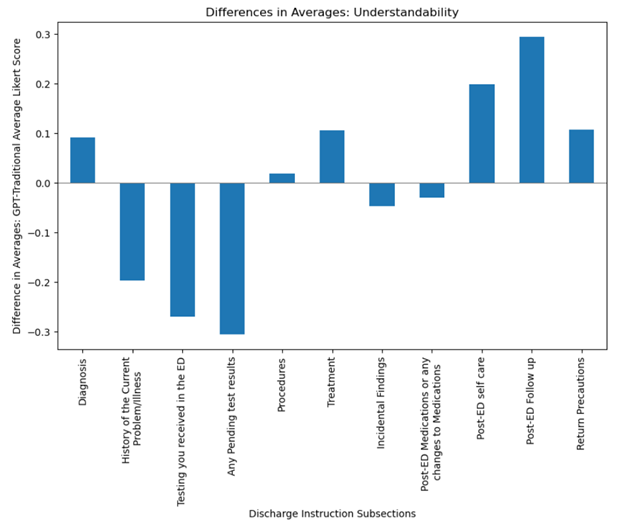

Supplement: Multimedia Appendix 4 [file jmir_v26i1e60336_app4.png]

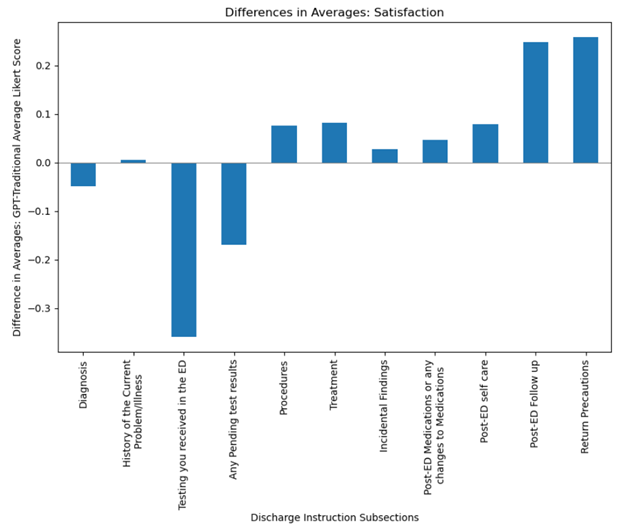

Supplement: Multimedia Appendix 5 [file jmir_v26i1e60336_app5.png]
